# Predicting first-grade mathematics achievement: the contributions of domain-general cognitive abilities, nonverbal number sense, and early number competence

**DOI:** 10.3389/fpsyg.2014.00272

**Published:** 2014-04-04

**Authors:** Caroline Hornung, Christine Schiltz, Martin Brunner, Romain Martin

**Affiliations:** ^1^Cognitive Science and Assessment, Education, Culture, Cognition and Society, University of LuxembourgLuxembourg, Luxembourg; ^2^Department for Educational Science and Psychology, Freie Universität BerlinBerlin, Germany

**Keywords:** longitudinal study, early number competence, working memory, nonverbal number sense, first-grade mathematics, kindergarten, structural equation modeling, mediation analyses

## Abstract

Early number competence, grounded in number-specific and domain-general cognitive abilities, is theorized to lay the foundation for later math achievement. Few longitudinal studies have tested a comprehensive model for early math development. Using structural equation modeling and mediation analyses, the present work examined the influence of kindergarteners' nonverbal number sense and domain-general abilities (i.e., working memory, fluid intelligence, and receptive vocabulary) and their early number competence (i.e., symbolic number skills) on first grade math achievement (i.e., arithmetic, shape and space skills, and number line estimation) assessed 1 year later. Latent regression models revealed that nonverbal number sense and working memory are central building blocks for developing early number competence in kindergarten and that early number competence is key for first grade math achievement. After controlling for early number competence, fluid intelligence significantly predicted arithmetic and number line estimation while receptive vocabulary significantly predicted shape and space skills. In sum we suggest that early math achievement draws on different constellations of number-specific and domain-general mechanisms.

## Introduction

Developing math proficiency is a central educational goal and a focus of national school curricula (e.g., Jordan et al., 2010). Nevertheless, math difficulties are typically recognized (too) late in elementary school, when children are already lagging behind their peers and obtaining poor math grades. Recent findings suggest that systematic early math education, screening and targeted intervention programs would allow young children at risk for math difficulties in later grades to be identified and supported at critical early stages(Jordan et al., 2009, 2010; Siegler and Ramani, 2009; Clark et al., 2013). In order to improve early educational practice in mathematics it is crucial to clearly identify the building blocks that should be targeted by these intervention programs. Longitudinal studies allow us to investigate which early abilities predict later math development in children (e.g., LeFevre et al., 2010). Thus, the present study tests a comprehensive model investigating how 5-year-olds' basic number-specific and domain-general abilities are related on the one hand to early number competence in kindergarten and on the other hand to math achievement in first grade.

We suggest that the interplay between basic number-specific and domain-general abilities leads to an early stage of formal number skills in kindergarteners, early number competence (ENC). ENC is also referred to in the literature as early numeracy skills (e.g., Kroesbergen et al., 2009; Aunio and Niemivirta, 2010) and symbolic number sense (Jordan et al., 2010). It refers to a range of conceptual knowledge and procedural skills that require the use of number words and symbols for exact number representations (see Gersten et al., 2005). It is a culturally acquired ability and may therefore depend on the development and the integration of multiple basic cognitive abilities (Geary, 1995; Bull and Espy, 2006; Kroesbergen et al., 2009; Passolunghi and Lanfranchi, 2012).

Evidence from a number-specific perspective suggests that math development is grounded in nonverbal number sense (Dehaene, 1997, 2009; Butterworth, 2005; Geary et al., 2008; Desoete et al., 2009; Libertus et al., 2011, 2012; Lourenco et al., 2012; Starr et al., 2013), already present in infancy (Wynn, 1992; Wynn et al., 2002; Feigenson et al., 2004; McCrink and Wynn, 2004; Starr et al., 2013). Past research has identified two nonverbal systems for tracking quantity, an exact number system and an approximate number system. The exact number system allows the fast and exact apprehension of small quantities up to 3 or 4 (i.e., subitizing) and supports the learning of the number sequence (Butterworth, 2005). Nonverbal number sense usually refers to the second system, the approximate number system (hereafter ANS). The ANS allows for the representation of magnitudes on a mental number line and the approximate discrimination of large quantities (Cantlon et al., 2009; also see Dehaene, 2009). The ANS refers to an ontogenetic and evolutionary foundation allowing approximate magnitude representations without symbolic number knowledge (Dehaene, 1997). According to this view, ANS acuity predicts children's math achievement (Gilmore et al., 2010; Reeve et al., 2012). The ANS is typically assessed with approximation number tasks requiring the participants to compare two sets of items and to choose the largest set. The performance on these tasks depends on the ratio between the magnitudes to compare. Discrimination of ratios closer to 1 is related to higher ANS acuity (Halberda et al., 2008). Research on mathematical learning disabilities (MLD) in young children repeatedly found typically developing children outperforming MLD children on magnitude comparison tasks (e.g., Desoete et al., 2012). This recent finding supports the conception of a “defective number module” resulting in lower ANS acuity in young MLD children (Landerl et al., 2004; Mussolin et al., 2010; Piazza et al., 2010). Prior research repeatedly found a relationship between ANS acuity and math performance in elementary school children and adults (e.g., Halberda et al., 2008; Gilmore et al., 2010). However, only few attempts were made to relate the ANS to formal early math skills in kindergarteners (Kroesbergen et al., 2009; Sasanguie et al., 2012; Mejias and Schiltz, 2013; Starr et al., 2013; Xenidou-Dervou et al., 2013).

To develop exact math competencies, nonverbal number sense may not be sufficient and may be assisted by domain-general abilities (Carey, 2004). Learning and academic achievement are supported by domain-general abilities such as working memory, (Jordan et al., 1995; Swanson and Kim, 2007; Alloway, 2009) and intelligence (Colom and Flores-Mendoza, 2007; Rohde and Thompson, 2007; Calvin et al., 2010).

Working memory (hereafter WM) is critical for numerous cognitive skills, including informal and formal math development (Noël, 2009; Friso-Van den Bos et al., 2013; see for a review Raghubar et al., 2010), reading comprehension (Daneman and Carpenter, 1980), and reasoning ability (Alloway and Alloway, 2010). According to Cowan (2008) WM refers to a set of domain-general mental resources facilitating the continuous interplay of storage and processing in everyday cognitive activities. Various WM models are plausible in children (Hornung et al., 2011): However developmental and educational studies usually refer to Baddeley and Hitch's multicomponent model, (1974). This WM model is composed of two passive temporary domain-specific stores (i.e., phonological loop and visuo-spatial sketchpad), and a general capacity-limited central executive. The phonological loop and visuo-spatial sketchpad briefly store information such as numbers, positions or intermediate results in verbal or visuo-spatial forms respectively. The central executive refers to executive processes allocating attentional resources to various processes such as updating, controlling, planning, and switching in order to complete a task. The central executive has been related to children's arithmetic skills (e.g., Bull and Scerif, 2001; in seven-year-old children) and counting (Kroesbergen et al., 2009; Noël, 2009, in 4-to-6-year-old children). These findings emphasize the role of the domain-general WM component in early math development (also see Passolunghi and Siegel, 2004; Mazzocco and Kover, 2007; Passolunghi et al., 2007; De Smedt et al., 2009). The roles of the verbal and visuo-spatial storage components are less conclusive in early math development and may depend on the tasks used and the characteristics of the study sample (e.g., age, cf. Friso-Van den Bos et al., 2013). De Smedt et al. (2009) found that first graders' visuo-spatial storage capacity was uniquely relevant to mathematical skills while verbal storage capacity was not (also see Bull et al., 2008; LeFevre et al., 2010). While Passolunghi and Lanfranchi (2012) found no significant relationship between specific storage components and early mathematics, they argued for the crucial role of executive processes.

Fluid intelligence, which commonly refers to reasoning ability, has been repeatedly related to later academic achievement and problem solving (Jensen, 1998), ENC (Kroesbergen et al., 2009) as well as later math achievement (Deary et al., 2007; Moenikia and Zahed-Babelan, 2010). Nevertheless until now research did not clarify the relationship between fluid intelligence and early math achievement.

Finally, language ability is necessary to develop ENC (Jordan et al., 2003; Donlan et al., 2007) because it allows the initial acquisition of the verbal integer list “one, two, three, four, … ” (Fuson, 1988; Wiese, 2007). Donlan and colleagues found that children with specific language impairments performed significantly lower in counting and calculation tasks than did typically developing children. These findings suggest that counting and calculation procedures draw at least partly on verbal routines. However, much less is known about the predictive power of kindergarteners' fluid intelligence and language ability on first grade math outcomes.

Considering the variety of factors potentially influencing early math development, it is not surprising that prior studies led to inconsistent findings on the respective role of number-specific and domain-general abilities in early math development. Halberda et al. (2008) related ninth graders' approximate number system acuity to their third grade general math achievement after controlling for 16 domain-general abilities. Others found on the contrary that number-specific and domain-general abilities both independently relate to early math development, such as kindergarteners' counting skills, (Kroesbergen et al., 2009), first graders' word problem solving (Fuchs et al., 2010a), enumeration, calculation, geometry, measurement and number line estimation (LeFevre et al., 2010), as well as third graders' general math achievement (Krajewski and Schneider, 2009). Finally, Noël (2009) emphasized the role of WM in kindergarteners' counting, but the study did not control for nonverbal number sense and other domain-general abilities. In a first effort to integrate various building blocks in a developmental model, LeFevre et al. (2010) recently proposed three distinct predictor pathways to explain various early math outcomes. Linguistic, quantitative and spatial attention predictors were assessed in kindergarten at the same measurement point. All three predictors uniquely contributed to kindergarteners' ENC concurrently assessed, as well as to various math outcomes assessed 2 years later in first grade. Spatial attention was assessed by a visuo-spatial WM task and related uniquely to six out of seven math skills (also see Bull et al., 2008; Krajewski and Schneider, 2009). The linguistic pathway was especially relevant for symbolic number processing and the quantitative pathway was especially relevant for magnitude comparison. Domain-general predictors such as executive processes (e.g., Noël, 2009; Clark et al., 2013) verbal short-term storage capacity (Swanson and Kim, 2007; Bull et al., 2008), and fluid intelligence (Kyttälä and Lehto, 2008) have contributed to math outcomes but were not yet included in an early math development model with concurrent number-specific predictors (cf., LeFevre et al., 2010). Recently, Passolunghi and Lanfranchi (2012) suggested that more longitudinal studies were needed that concurrently administered predictor variables potentially contributing to explain math outcomes. In sum, the respective roles of number-specific and domain-general abilities to math development currently remain tentative. On the one hand this is possibly due to the range of cognitive precursors and math outcomes assessed and the specific analyses conducted. On the other hand inconsistencies may also be due to differences in terms of participants' age and educational experience across studies. Regrettably few attempts have been made to model early math development in 5-year-olds before formal schooling. Such an approach would be particularly promising in order to (a) test theoretical frameworks, (b) study the relations between specific and general cognitive predictor abilities and early math competencies, (c) better understand the typical and atypical pathways in early math development in order to detect the kind of difficulties of a low performing child, and consequently (d) improve educational practices in kindergarten and first grade.

To the best of our knowledge there have been no longitudinal studies investigating the relationship between approximate number abilities, general cognitive abilities, and early symbolic number skills in kindergarten and math achievement in first grade. The present study complements and extends previous work by using a longitudinal approach to investigate how number-specific and domain-general cognitive abilities in kindergarten are interrelated and predict early math achievement in first grade (e.g., Espy et al., 2004; Kroesbergen et al., 2009; Noël, 2009; LeFevre et al., 2010). Seven cognitive predictor measures are tested in this study: nonverbal number sense, verbal and visuo-spatial short-term storage capacity, WM capacity, fluid intelligence, language comprehension, as well as ENC. ENC is considered as mediator, because it can be considered to be a first outcome of early forms of formal math training even if this training happens at least partly in informal contexts.

While a series of studies indicate that ENC is key for developing later math competencies the respective roles of nonverbal number sense and domain-general abilities are still less clear when assessed concurrently (Aunola et al., 2004; Gersten et al., 2005; Jordan et al., 2006, 2007, 2009, 2010; Krajewski and Schneider, 2009; Landerl and Kölle, 2009).

Thus, the present study investigates two main hypotheses. First, we suggest that nonverbal number sense and domain-general abilities uniquely relate to ENC in kindergarten (see also Kroesbergen et al., 2009; LeFevre et al., 2010). We expect that both number-specific and general predictors critically influence math development at this early stage (see also Mejias and Schiltz, 2013). Second, we contend that ENC in kindergarten is the key building block for formal mathematics and therefore ENC becomes a critical mediator in the relationship between kindergarten number-specific and domain-general predictors and first grade math outcomes. Thus, we concurrently assessed 7 key predictors for first grade math in order to distinguish their respective roles in early math development. To test our hypotheses we capitalize on structural equation modeling (SEM) and mediation analyses. By taking full advantage of SEM and our specific set of measures, the present study significantly extends the current understanding of the contribution of number-specific and domain-general abilities to ENC in kindergarten and math achievement in first grade.

## Material and methods

### Participants

This longitudinal study involved two measurement points: the first when participants were in their second year of kindergarten in Luxembourg (5 to 6-years-olds) and the second 12 months later at the end of first grade. All participating children were taught the same mathematics curriculum for 8 months in first grade. While no formal math was taught in kindergarten, first grade math mainly concentrated on basic arithmetic (i.e., additions and subtractions) in the number range from 0 to 20, on shape and space skills (completing visual patterns, identifying shapes) and the symbolic number line (e.g., write numbers on a line with 0–20 interval and interim markers). The present analyses are based on the data obtained for all 165 children (50.9% boys) who participated at the first point of measurement (for handling of missing data, see the statistical analyses section below). Written parental consent was obtained for all participating children. Their mean age was 74.8 months in kindergarten (*SD* = 3.80 months). All children spoke and understood Luxembourgish. Sixty-four percent of the children were bilinguals and spoke a second language at home. This multilingual situation is typical for public schools in Luxembourg. The practice examples revealed that all the children understood the task instructions. Children's socio-economic background (as measured by a parent questionnaire assessing occupation) was diverse: 34.8% of mothers and 51.9% of fathers were blue-collar workers; 20% of mothers and 23.1% of fathers were employed in the private sector; 11.6% of mothers and 13.5% of fathers worked for the government or in the public sector; 5.2% of mothers and 9% of fathers were self-employed; 1.9% of the mothers and 1.2% of the fathers were craftswomen and craftsmen, respectively; 26.5% of mothers and 1.3% of fathers were not employed.

### Procedure

In kindergarten, the participating children were tested individually in a quiet room in two separate test sessions, each lasting approximately 35 min, at an interval of 3 h–4 days. Exceptions were made for three children who participated in the second session 1 week later due to sickness. The tasks were presented in a fixed order as follows. In the first session two WM components (phonological loop and central executive), nonverbal number sense and ENC were assessed. In the second session the third WM component (visuo-spatial sketch pad), fluid intelligence and receptive vocabulary were assessed. The task instructions were brief and standardized. The experimenter was the same for all children and test sessions. Children were not given any performance-contingent feedback during the test sessions. With the exception of the verbal counting scale and the receptive vocabulary task, all tasks were presented on a portable computer.

One year later, at the end of first grade three math outcomes were assessed, related to the national curriculum. The testing session took approximately 1 h and was held in the classroom with the complete group of students. The experimenter was the same for all the participants. The classroom teacher or an additional research assistant was present during the test session to assist the participants if needed and to prevent copying. First, children performed a speeded standardized mathematics test, HRT 1–4, (Heidelberg mathematical test for Grade 1–4); (Haffner et al., 2005). This test assessed two different competencies, arithmetic and shape and space skills. After the speeded mathematics test the participants completed a number line estimation task without time constraints.

### Standardized measures administered in kindergarten

As recommended by Fuchs and colleagues, we used a broad range of measures to predict math outcomes 1 year later in order to minimize specification errors that would produce biased estimates of the effects of a single predictor (Pedhazur and Schmelkin, 1991; Fuchs et al., 2010b, in Floyd et al., 2003). Appendix A presents all the tasks and tests administered, as well as example items.

#### Early number competence

We administered verbal counting, dot counting, and Arabic number comparison to measure ENC in kindergarten.

***Verbal counting***. *Fifteen* trials taken from the Diagnostic Tests for Metacognitions and Mathematics (Salonen et al., 1994 in Aunola et al., 2004)assessed verbal counting skills. Participants were required a) to count up to 50 (3 points), b) to count up from a number (4 points), c) to count backward from a number (4 points), and d) to count x steps from a given number (4 points, “What number do you get if you count three steps from 6?”). One point was awarded for each correct answer resulting in a maximum score of 15. Cronbach's Alpha was 0.86.

***Dot counting***. In 15 trials, children were presented arrays of dots (numerosities: 4–9) on a portable computer screen. Two to three trials were presented per numerosity. On half of the trials the arrays were linear and on half of the trials the dots were randomly scattered over the screen. On each trial, the children had to count the dots and say how many dots were on the screen. The experimenter encoded the children's answers by pressing the corresponding digit. One point was awarded for each correct answer resulting in a maximum score of 15. Cronbach's Alpha was 0.73.

***Arabic number comparison***. The Arabic number comparison task was drawn from the TEDI-Math Battery (Van Nieuwenhoven et al., 2001) and was computer-assisted with PsyScope. Test items were 11 pairs of Arabic numbers: three pairs of single-digit, six pairs of two-digit, and two pairs of three-digit numbers. Two numbers appeared simultaneously on the screen, one on the left, and one on the right. The experimenter did not read the presented numbers. After visual presentation the child had to judge which number was larger in numerosity and press the corresponding response button on the left (pink button) or the right (blue button) of the keyboard. The answers were automatically encoded. The task produced a poor statistical reliability (α = 0.42) and we thus omitted four items to improve the reliability (i.e., both trials with three-digit numbers and two trials with incongruent number information e.g., 42–38). The remaining items were three pairs of single digit numbers and 4 pairs of two-digit numbers. Cronbach's Alpha of the modified score was 0.70. One point was awarded for each correct answer, yielding a maximum score of 7. Cronbach's Alpha of the total score across all ENC tasks was 0.76.

#### Working memory (WM)

WM components were assessed by two verbal short-term storage tasks, two visuo-spatial short-term storage tasks, and two verbal working memory tasks, each beginning with 3 practice trials at a list length of two stimuli. Each increase in list length was announced to the participant to reduce the effect of attentional factors on task performance. The auditory items of the verbal span tasks were digitally recorded by a female speaker to enhance standardized test procedure and inserted into four PowerPoint presentations, each corresponding to a distinct verbal span task. Children performed the verbal span tasks orally, and the experimenter wrote down their answers. The visuo-spatial span tasks were developed using the Quest Net multimedia authoring system (Allen Communication, 1996; for a similar task design in adults see Martin et al., 2008).

***The phonological loop***. We administered the digit recall and the pseudo-word recall tasks to evaluate the phonological loop. While the digit recall relies on the storage of number-specific information, pseudo-word recall is a pure verbal measure of verbal short-term storage because the unfamiliar sequences of syllables are unlikely to be supported by long-term memory representations (Gathercole et al., 1994). The digit recall task was translated from the WM Test Battery for Children (WMTB-C, Pickering and Gathercole, 2001). Participants recalled a list of single digits presented at a 1 s interval in the same order directly after presentation. The task starts with 6 trials of two digit sequences and progresses to sequences of three to six digits. Six trials were presented for each span list; a child correctly recalling three successive trials in a span list moved on to the next span list. Cronbach's Alpha was 0.90. The procedure of the pseudo-word recall task was identical to that of the digit recall task. The only difference was that participants had to recall lists of syllables in the order given (e.g., “MI FA LU RO”). Cronbach's Alpha was 0.90. Because no individual differences were found on span lists 2 and 6 in either verbal short-term storage task, we excluded these data from the analysis. The score on the verbal short-term storage tasks corresponded to the sum of correct trials on span lists 3, 4, and 5 (maximum score = 18).

***Central executive***. We administered the backward digit recall and the backward color recall to evaluate the central executive: In the backward digit recall task, participants recalled a list of single digits in reverse order. In the backward color recall task, participants recalled a list of color words in reverse order. Cronbach's Alpha was 0.84 for both tasks. The score on the verbal working memory capacity tasks corresponded to the sum of correct trials on span lists 2, 3, and 4 (maximum score = 18).

***Visuo-spatial sketchpad***. We administered two dynamic location span tasks (grid recall / no grid recall) to assess the visuo-spatial sketchpad. Both tasks were presented on a tablet PC screen. Children had to memorize and reproduce the sequential positions of a dwarf that appeared and disappeared again shortly afterwards. They used a pen to indicate the remembered locations on the touch-sensitive computer screen. Participants' answers were automatically scored. The task began with two practice trials and consisted of two blocks of 11 test trials, each block comprising three trials with two, four trials with three, and four trials with four targets. The test trials were presented in fixed order in two distinct conditions that were alternated randomly from trial to trial. In the first condition, targets appeared and disappeared in a visible grid on the screen. The grid had four columns and four rows, yielding a total of 16 cells. In the second condition, targets appeared and disappeared on a black screen (no grid). The child had to rely on features other than the grid to find the hidden targets. The cognitive demands of the two conditions differed: the no grid condition required higher executive control processes to actively structure the screen and to encode and recall the hidden targets (Hornung et al., 2011). Accordingly, the mean scores showed that children's performance was considerably higher in the grid condition than in the no grid condition, the more difficult attention-demanding condition (see Table 1). Cronbach's Alpha was 0.72 for the test items in both conditions. The maximum possible score in each visuo-spatial span recall task was 11.

**Table 1:** **Intercorrelations and descriptive statistics for number-specific, domain-general ability and math competency measures**.

**Measures**	**1**	**2**	**3**	**4**	**5**	**6**	**7**	**8**	**9**	**10**	**11**	**12**	**13**
**ADMINISTERED IN KINDERGARTEN**													
**Working memory (WM)**													
1. Central executive	–												
2. Phonological loop	**0.58**	–											
3. Visuo-spatial sketchpad Number sense	**0.35**	0.17	–										
4. Dot comparison	**0.37**	0.21	**0.31**	–									
5. Stick comparison Fluid intelligence	**0.43**	**0.30**	**0.31**	**0.60**	–								
6. Raven's CPM (set A, AB, B)	**0.48**	**0.36**	**0.40**	**0.33**	**0.39**	–							
Receptive Vocabulary													
7. BPVS	**0.51**	**0.40**	**0.36**	**0.35**	**0.44**	**0.47**	–						
Early number competence													
8. Verbal counting scale	**0.68**	**0.49**	**0.39**	**0.45**	**0.45**	**0.53**	**0.57**	–					
9. Dot counting	**0.42**	0.24	**0.27**	0.25	**0.33**	0.20	**0.28**	**0.41**	–				
10. Arabic number comparison	**0.49**	**0.38**	**0.43**	**0.38**	**0.38**	**0.40**	**0.40**	**0.64**	**0.38**	–			
**ADMINISTERED IN FIRST GRADE**													
**Math assessment**													
11. Arithmetic	**0.58**	**0.46**	**0.36**	**0.34**	**0.33**	**0.54**	**0.53**	**0.78**	**0.32**	**0.47**	–		
12. Shape and space skills	**0.54**	**0.43**	**0.37**	0.27	**0.39**	**0.40**	**0.55**	**0.63**	**0.30**	**0.45**	**0.67**	–	
13. Number line estimation^a^	**0.52**	**0.32**	**0.33**	**0.36**	**0.38**	**0.56**	**0.52**	**0.60**	0.20	**0.46**	**0.67**	**0.57**	–
Percentage of missing values	0	0	1.2%	0.6%	0.6%	0.6%	1.2%	0	0	0	8.5%	8.5%	18.2%
Mean	7.04	7.25	4.19	17.96	31.81	19.19	59.77	7.78	12.05	5.57	8.74	6.95	0.17
*SD*	4.10	3.85	3.23	3.01	2.19	4.26	23.68	4.07	2.74	1.65	3.35	3.74	0.09
Skewness	−0.08	0.39	0.26	−1.38	−0.68	0.14	0.14	0.21	−1.23	−0.55	0.14	0.41	0.76
Kurtosis	0.29	0.04	−0.41	1.59	−0.39	−0.44	−1.05	−0.86	1.10	0.21	−0.12	−0.28	−0.06

Correlations are all reaching significance at the corrected.05 alpha levels (p-adjusted values, R. Core Team, 2013). Correlations reaching significance at the corrected 0.001 alpha level are in bold. CPM, Colored Progressive Matrices; BPVS, British Picture Vocabulary Scale.

a The number line estimation scores are absolute error scores; to avoid negative numbers, the correlations are absolute values.

#### Nonverbal number sense

Nonverbal number sense was assessed by two magnitude comparison tasks: a dot comparison task and a stick comparison task. In creating task stimuli, we controlled for visual cues in order to prevent children from relying on perceptual factors when comparing sets of magnitude (Mix et al., 2002; Abreu-Mendoza et al., 2013). Rousselle et al. (2004) found that the use of diverse visual controls affected children's performance in approximate magnitude comparisons. We therefore chose density control (easy control) and total filled area control, which constrains children to rely on number cues. Magnitudes (i.e., from 3 to 30) were presented in pseudorandom order in both tasks and four different numerical ratios were used (i.e., 1:2; 2:3; 3:4; 4:5). Children were instructed to respond as fast and accurately as possible without counting. Both tasks were timed and children pressed either a pink (left) or a blue (right) button to identify the side of the screen with the larger magnitude. Each test item was presented for a maximum of 4000 ms. If a child did not manage to respond within this time window, the child obtained zero points. This procedure was used to compel children to rely on their approximate sense for quantities and avoid counting. Children received one point for each correct response. Both tasks started with two practice trials. During practice the experimenter gave performance-contingent verbal feedback.

***Dot comparison***. Two sets of dots were simultaneously presented on a portable computer screen. Children were asked to select the set containing more dots. For each of the 11 pairs, the larger magnitude appeared once on the left side and once on the right side, yielding a total of 22 trials. Stimuli were pairs of white squares with a black border containing a variable number of black dots of equal size. A black line separated both squares. To control for density in the dot comparison task, the same space was kept between the dots. Initially, 21 pairs of dots were presented. However, we eliminated 10 pairs because these items were not accurately controlled in terms of density. Cronbach's Alpha for the final test items was 0.70.

***Stick comparison***. Two sets of sticks were simultaneously presented on a computer screen. Children were required to select the set containing more sticks. For each of the 21 pairs, the larger magnitude appeared once on the left side and once on the right side, yielding a total of 42 trials. Stimuli were pairs of white squares with a black border containing a variable number of black sticks (vertical rectangles). Both squares were presented on a white background and were separated by a fixation cross (distance between squares = 15 mm). The total filled area of the stick sets was controlled. We equated the area occupied by the sticks by reducing the total size of the sticks in larger sets. We avoided constellations in which the set with the larger number of sticks was also the set with smaller sticks by using sticks of different heights and widths and ensuring that the overall area occupied by the smaller and larger sticks was the same in both sets. Cronbach's Alpha was 0.82. Since the density control appears to be an easier visual control than the total surface area control, we suggest that the stick comparison task may be more strongly related to ENC, especially dot counting.

#### Receptive vocabulary

The first 10 test sets of the British Picture Vocabulary Scale (Dunn et al., 1997) were administered to evaluate children's receptive vocabulary. Each test set comprised 12 trials. On each trial, the experimenter showed the child four pictures and presented a word verbally in Luxembourg language. The child had to point to the picture corresponding to that word. The test sets (from 1 to 10) increased in difficulty (i.e., the words to recognize became less familiar objects). Test set 1 was used as a practice set. For structural equation modeling, we parceled the nine remaining test sets into three verbal comprehension test scores measuring the latent construct of receptive vocabulary. We balanced the parcels in terms of difficulty (see Little et al., 2002). Verbal comprehension test score 1 represented the aggregated scores from test sets 2, 5, and 8. Test score 2 represented the aggregated scores from test sets 3, 6, and 9 and test score 3 represented the aggregated scores from test sets 4, 7, and 10. Parceling has particular psychometric advantages in small sample sizes, because fewer parameters are necessary to specify a latent construct (Little et al., 2002). Cronbach's Alpha was 0.95.

#### Fluid intelligence

We measured fluid intelligence using a widely applied abstract reasoning ability test, Raven's Colored Progressive Matrices (Raven et al., 1998). The test was presented in PowerPoint format on a portable computer, and the experimenter entered the children's answer to each item on a response sheet. The test items were abstract shapes and patterns, from each of which a piece was missing. Six response options were offered below the test item. The children were instructed to choose the one that fit into the blank space. The test was composed of three sets (A, AB, and B), each comprising 12 items. All children completed the three parts of the test; their score on each part corresponded to the number of correctly solved items (up to a maximum score of 12 per set). The maximum possible score for fluid intelligence was 36. Raw scores obtained for each set were used for statistical analyses. Cronbach's Alpha was 0.74.

### Standardized measures assessing first grade math outcomes

In first grade, we assessed three math outcomes in line with Luxembourg's national curriculum: arithmetic, shape and space skills, and number line estimation. All tasks began with several practice items. Arithmetic and shape and space skills were assessed by tasks taken from a standardized speeded test, the Heidelberg Mathematics Test (Haffner et al., 2005). Each task started with four practice items. Henceforth children had 2 min to complete each of the speeded math tasks.

#### Arithmetic

More specifically, arithmetic was measured by five tasks: mental addition (e.g., 1 + 6 = _), mental subtraction (e.g., 4 – 1 = _), number comparison (e.g., 11 _ 12), basic equations (e.g., 5 + __ = 7 + 2), and number sequences (e.g., 1 2 1 2 1 2 1 2 _ _ _). Children received one point for each correctly solved item. To correct for guessing in the Arabic number comparison task, in which there were only three answer options (<, >, =), we complied with the test developers and subtracted one point from a child's score on this task for each incorrect answer. A sum score of general arithmetic ability was then computed across all five tasks for use in the raw correlational analyses. Cronbach's Alpha was 0.87.

#### Shape and space skills

Shape and space skills were assessed by two tasks: length estimation and counting blocks. In the length estimation task the child had to estimate the length (number of steps) of a series of presented black lines by comparing each line with three bolder black lines presented on the top of the test sheet. The three bold lines corresponded respectively to 1, 5 or 10 steps. In the counting blocks task the child had to find out how many blocks build a presented figure. To provide the correct answer, the child not only had to count the visible blocks but also visualize and count those that were completely or partly covered and yet essential to build the figure. A sum score for both tasks was computed for use in the raw correlational analyses. Cronbach's Alpha was 0.86.

#### Number line estimation

We used a paper-and-pencil number line estimation task, which taps children's approximate mental representation of numbers and thus provide an indicator of developing symbolic number sense (see Schneider et al., 2009; Siegler, 2009). In this task, the children had to place a number on a physical number line with fixed endpoints (0–100) without interim numbers or marks, by marking the line with a cross. The task consisted of 17 test items (90, 50, 30, 40, 25, 5, 15, 80, 69, 48, 70, 95, 14, 60, 39, 33, 19) and had no time constraint. Before the test, the experimenter explained the task with one practice item on the blackboard. We calculated the percent absolute error (PAE) to score the children's performances for each estimated number (Siegler and Booth, 2004):

PAE = [(child's estimate- number to be estimated)/number line scale]

For example, if the number to be estimated on the 0–100 number line was 25, and the child made a mark at the position that corresponds to 50, PAE would be 0.25 for this number [i.e., (50–25)/100 = 0.25; corresponding to 25% of absolute error]. From these calculations, mean PAE scores could be computed. We divided the task items of the number line estimation into three parcels for defining the construct in structural equation modeling (Little et al., 2002). Parcel 1 represented the average PAE score for the 6 following test items (90, 40, 15, 48, 14, 33), parcel 2 represented the average PAE score for test items (50, 25, 80, 70, 60, 19) and parcel 3 represented the average PAE score for the test items (30, 5, 69, 95, 39). Cronbach's Alpha was 0.83.

### Statistical analyses

Structural equation modeling (SEM) is a promising statistical method because it reduces measurement error and allows estimating the fit of a theoretical mediation model. Thus, it offers several advantages relative to traditional mediation analysis with regression models. According to Bollen (1989), the variance common to a set of related measures gives a more coherent representation of an underlying theoretical construct than any single measure of a task as task-specific variance is not represented in the variance of the latent construct (given that a broad range of measures is applied). Moreover, latent variables are free of measurement error. That is to say, relative to traditional regression analyses, SEM provides an unbiased estimate of the longitudinal and mediation relationships between cognitive precursors, ENC (the mediator), and math outcomes (cf. Cohen et al., 2003). And, SEM allows us to estimate the fit of a theoretical mediation model to the data (Cohen et al., 2003). Finally, SEM allows us to compute confidence intervals and to judge the statistical significance of mediation effects (MacKinnon et al., 2007).

**Figure 1:**
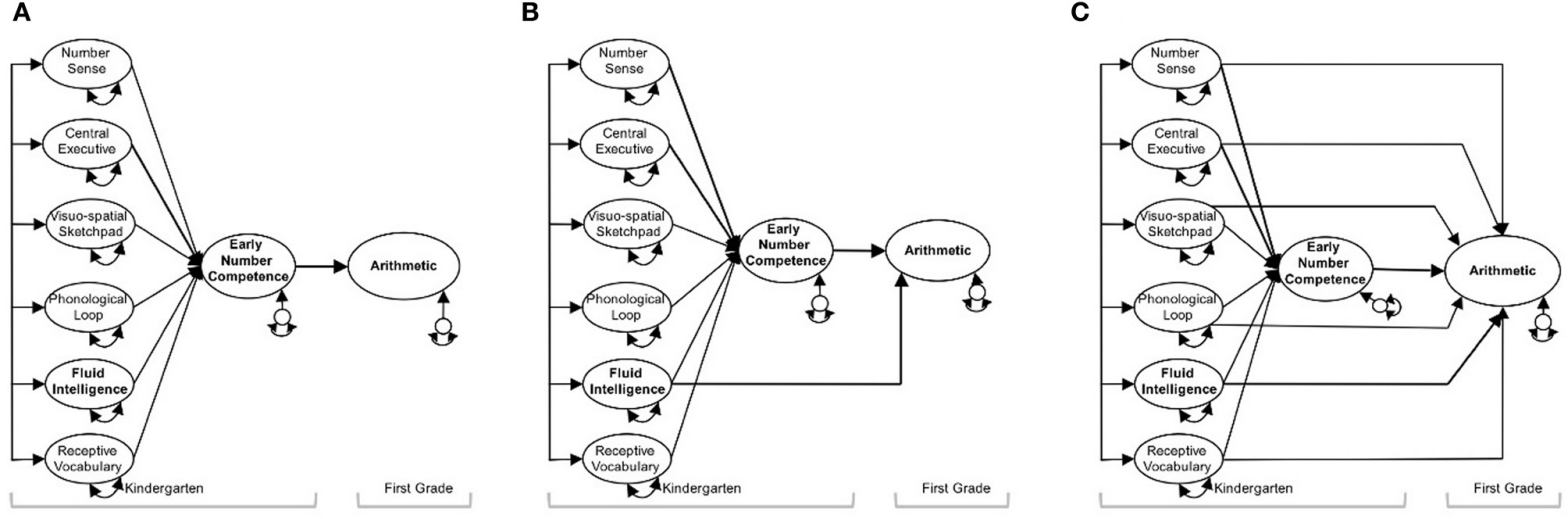
**Illustration for mediation analyses**. Three mediation models to study how cognitive predictors measured at kindergarten influence arithmetic in first grade via early number competence.

Given these advantages, we run a total of nine structural models to study whether and to what extent ENC is a critical mediator of the effects of kindergarteners' transversal domain-general and domain-specific basic cognitive abilities (i.e., nonverbal number sense, WM components, fluid intelligence and receptive vocabulary) when predicting three different math outcomes 1 year later. Specifically, the first part of each model investigates how kindergarten predictor measures (i.e., the independent variables) contribute to ENC (i.e., the mediator), the second part of each model tests how all measures taken in kindergarten (i.e., independent variables and mediator) affect a specific math outcome 1 year later (i.e., arithmetic, shape and space skills, and number line estimation). In general, we estimate three mediation models for each of the three math outcomes at first grade: a complete mediation model, a partial mediation model and a saturated structural model. In the complete mediation models (Model a, d, and g), the relations between kindergarten precursors and specific math outcomes in first grade were completely mediated by ENC. By contrast, in the partial mediation models (Model b, e, and h) the relations between kindergarten predictors and math outcomes were not solely mediated by ENC, but rather we extend the complete mediation models by including only those direct effects that reached statistical significance. Finally, in the fully saturated models (Models c, f, and i) we allowed for direct effects of all kindergarten predictors on math outcomes in addition to the indirect effects via ENC. By comparing the partially mediated models with the complete mediation models we learn whether ENC completely mediates the effects of basic cognitive predictors on math outcomes. By comparing the fully saturated models with the partial saturated models we learn whether additional direct effects are necessary to account for the relations among basic cognitive predictors, ENC, and math outcomes. Taken together, these model comparisons are necessary to paint a differentiated and methodologically sound picture (MacKinnon et al., 2007; O'Boyle and Williams, 2011) whether and to what extent ENC operates as a critical mediator of the effects of domain-specific and domain-general cognitive predictors on first grade math outcomes. Figure 1 illustrates the mediation Models a, b and c for arithmetic.

We used the full information maximum likelihood (FIML) method with Mplus 5.2 (Muthén and Muthén, 1998–2007) to handle missing data in all analyses (Schafer and Graham, 2002; Newman, 2003; Baraldi and Enders, 2010). The percentage of missing values per variable are indicated in Table 1 ranging from 0% on the verbal span tasks to 18.2% on the number line estimation task. We assessed model fit using the χ^2^ goodness-of-fit statistic, the comparative fit index (CFI), the standardized root-mean-square residual (SRMR), and the root-mean-square error of approximation (RMSEA). Additionally, we used the root-mean-square error of approximation with a focus on the path model (RMSEA-P). This index assesses the fit of the relations among latent variables and outperforms the traditional composite fit indices in detecting model misspecification among latent variables (Williams and O'Boyle, 2011). A non-significant χ^2^ goodness-of-fit statistic indicates an adequate fit. The corresponding probability value indicates the probability of finding a multivariate difference of a certain size between the specified model and the sample data, given that the specified model is the “true” model in the population. CFI values larger than 0.95 and SRMR values below 0.08 indicate a good fit (Hu and Bentler, 1999). RMSEA values below 0.05 indicate a good model fit, values between 0.05 and 0.08 are a reasonable model fit (Browne and Cudeck, 1993), and values greater than 0.10 are a poor approximation of the model (Jöreskog, 1993). The same criteria apply to the RMSEA-*P* values (O'Boyle and Williams, 2011; Williams and O'Boyle, 2011). To judge the statistical significance of the mediation effects via ENC we followed MacKinnon (2008) and computed confidence intervals by means of a bias-corrected bootstrap method as implemented in Mplus 5.2.

## Results

Descriptive statistics and intercorrelations of all indicators of children's cognitive abilities are presented in Table 1. All measures met standard criteria of univariate normality with skewness for all measures below 3 and kurtosis for all measures below 4 (Kline, 1998). None of the raw correlations was above 0.80, suggesting that multicolinearity was not a problem in the present data at the measurement level (Kline, 1998). Several correlations are worth mentioning. All three WM components were positively correlated, although the correlation between the verbal and visuo-spatial storage systems was not significant (*r* = 0.17). The central executive correlated significantly with the phonological loop (*r* = 0.58) and with the visuo-spatial sketchpad (*r* = 0.35). Both, the central executive and the phonological loop were assessed by verbal span measures, which may explain their stronger association. Second, ENC measures were more strongly related to the central executive (*r* = 0.42–0.68) than to all other measures taken at kindergarten. Third, when comparing the relationships between nonverbal number sense and ENC measures, we found that dot counting accuracy was stronger related to stick comparison (total filled area control; *r* = 0.33) than to dot comparison (density control; *r* = 0.25). Fourth, all three first grade math outcomes were more highly correlated with the verbal counting scale (e.g., *r* = 0.78 with arithmetic) than with any other measure taken at kindergarten.

### Structural equation modeling and mediation analyses

Our aim was to study the relations between distinct domain-specific and domain-general abilities involved in early math development in kindergarten and first grade by using mediation analyses and structural equation modeling (SEM). Latent correlations for all cognitive abilities are presented in Table 2. The fit of this latent model including all variables was adequate (*x*^2^ = 376.42; *df* = 279; CFI = 0.97, RMSEA = 0.046; SRMR = 0.05).

**Table 2:** **Latent correlations between factors**.

**Latent correlations**	**1. (CE)**	**2. (PL)**	**3. (VS)**	**4. (NS)**	**5. (GF)**	**6. (RV)**	**7. (ENC)**	**8. (AR)**	**9. (SS)**	**10. (NL)**
1. Central executive (CE)	–									
2. Phonological loop (PL)	**0.67**	–								
3. Visuo-spatial sketchpad (VS)	**0.44**	0.26	–							
4. Number sense (NS)	**0.55**	**0.34**	**0.49**	–						
5. Fluid intelligence (GF)	**0.60**	**0.47**	**0.54**	**0.53**	–					
6. Receptive vocabulary (RV)	**0.55**	**0.48**	**0.41**	**0.51**	**0.55**	–				
7. Early number competence (ENC)	**0.79**	**0.58**	**0.52**	**0.63**	**0.66**	**0.61**	**–**			
8. Arithmetic (AR)	**0.65**	**0.55**	**0.41**	**0.49**	**0.69**	**0.55**	**0.86**	**–**		
9. Shape & space skills (SS)	**0.75**	**0.64**	**0.52**	**0.57**	**0.62**	**0.71**	**0.84**	**0.90**		
10. Number line estimation (NL)	**0.57**	**0.36**	**0.37**	**0.50**	**0.72**	**0.56**	**0.69**	**0.74**	**0.75**	**–**

All correlations were significant at the 0.001 level (except the correlation between PL and VS). The fit of this model was adequate: x^2^ = 376.42; df = 279; CFI = 0.97, RMSEA = 0.046; SRMR = 0.05.

Standardized parameter estimates are shown in Table 3. Note that all latent variables were well defined as indicated by the substantial and statistically significant factor loadings of each measure on the corresponding construct/factor.

**Table 3:** **Standardized parameter estimates**.

**Factor**	**Measure**	**Factor loading**	***SE***
Central executive	Backward color	0.85	0.03
	Backward digit	0.89	0.03
Phonological loop	Pseudo word	0.71	0.05
	Forward digit	0.96	0.04
Visuo-spatial sketchpad	Visuo-spatial span (grid)	0.76	0.06
	Visuo-spatial span (no grid)	0.79	0.06
Number sense	Dot comparison	0.81	0.05
	Stick comparison	0.81	0.05
Fluid intelligence	CPM A	0.57	0.07
	CPM AB	0.72	0.06
	CPM B	0.69	0.06
Receptive vocabulary	BPVS A	0.96	0.01
	BPVS B	0.98	0.01
	BPVS C	0.97	0.01
Early number skills	Counting scale	0.88	0.03
	Dot counting	0.50	0.06
	Number comparison	0.71	0.05
Arithmetic	Additions	0.85	0.03
	Subtractions	0.78	0.04
	Number comparisons	0.85	0.03
	Number equations	0.77	0.04
	Number sequences	0.67	0.05
Shape and space skills	Cubes	0.62	0.06
	Lengths estimation	0.73	0.06
Number Line Estimation	Number line estimation error rate A	0.81	0.04
	Number line estimation error rate B	0.84	0.03
	Number line estimation error rate C	0.89	0.03

These are the parameter estimates for the latent correlation model (see Table 2). CPM, colored progressive matrices; BPVS, British picture vocabulary scale.

Nonverbal number sense, receptive vocabulary and domain-general abilities were highly related to ENC in kindergarten and math outcomes in first grade, indicating that basic cognitive abilities (i.e., independent variables) contribute to ENC in kindergarten (i.e., mediator) and math outcomes in first grade (i.e., dependent variables), but probably to different degrees. ENC correlated highly with math outcomes in first grade. According to Baron and Kenny (1986) this correlational pattern between precursors, mediator and outcomes is a prerequisite for conducting mediation analyses.

We then analyzed mediation models for each of the three first grade math outcomes. For all structural models the estimation procedures converged, no parameter estimates were out of the range of admissible parameter estimates (e.g., negative variances), and all matrices of parameter estimates were positive definite. Importantly, the overall fit of the nine models was good; except Model g fit the data poorly according to the RMSEA_P index (0.107) and some residual correlations exceeding 0.1 (McDonald, 2010). Fit indices, model parameters and chi-square difference tests are reported in Table 4. Note that the factor loadings of cognitive measures on corresponding constructs in Models a to i differed only marginally from those reported in Table 3.

**Table 4:** **Mediation models to study how kindergarteners' abilities affect math outcomes in first grade via early number competence: fit statistics, parameters and chi-square difference tests**.

**Model**	**Model fit**							**χ^2^difference test**				
	***χ^2^***	***df***	***p***	***CFI***	***RMSEA***	***SRMR***	***RMSEA-P***	***R^2^***	***Residual correlations* (range of absolute values)**	***χ^2^_diff_***	***df_diff_***	***p***
**ARITHMETIC**												
a. Complete mediation model	268.19	187	0.001	0.97	0.05	0.05	0.06	0.74	(0–0.04)			
b. Partial mediation model	264.04	186	0.001	0.97	0.05	0.05	0.01	0.74	(0.01–0.05)			
Model b. vs. Model a.										4.15	1	0.04
c. Saturated structural model	259.15	181	0.001	0.97	0.05	0.05	0	0.79	(0–0.02)			
Model c. vs. Model b.										4.89	5	0.43
**SPACE AND SHAPE SKILLS**												
d. Complete mediation model	168.34	130	0.01	0.98	0.04	0.04	0.04	0.84	(0–0.10)			
e. Partial mediation model	162.74	129	0.02	0.98	0.04	0.04	0.06	0.83	(0–0.05)			
Model e. vs. Model d.										5.6	1	0.02
f. Saturated structural model	160.55	124	0.02	0.98	0.04	0.04	0	0.81	(0–0.03)			
Model f. vs. Model e.										2.19	5	0.82
**NUMBER LINE ESTIMATION**												
g. Complete mediation model	203.38	148	0.001	0.97	0.05	0.05	0.107	0.51	(01–0.21)			
h. Partial mediation model	192.34	147	0.01	0.98	0.04	0.04	0.04	0.58	(0–0.07)			
Model h. vs. Model g.										11.04	1	<0.01
i. Saturated structural model	185.95	142	0.01	0.98	0.04	0.04	0	0.64	(0.01–0.03)			
Model i. vs. Model h.										6.39	5	0.27
Cutoff criterion			>0.05	>0.95	<0.08	<0.08	<0.08		<0.10			

χ^2^, chi-square goodness-of-fit statistic; df, degrees of freedom; CFI, comparative fit index; RMSEA, root-mean-square error of approximation; RMSEA-P, RMSEA, with a focus on the path model; SRMR, standardized root-mean-square residual; R^2^, overall variance explained in mathematics outcome measured in first-grade by the independent variable(s); _diff_, difference. All models met the cutoff criteria for an acceptable to good fit, except Model g had an unacceptable fit according to the RMSEA-P cutoff criteria and the range of residual correlations.

**Figure 2:**
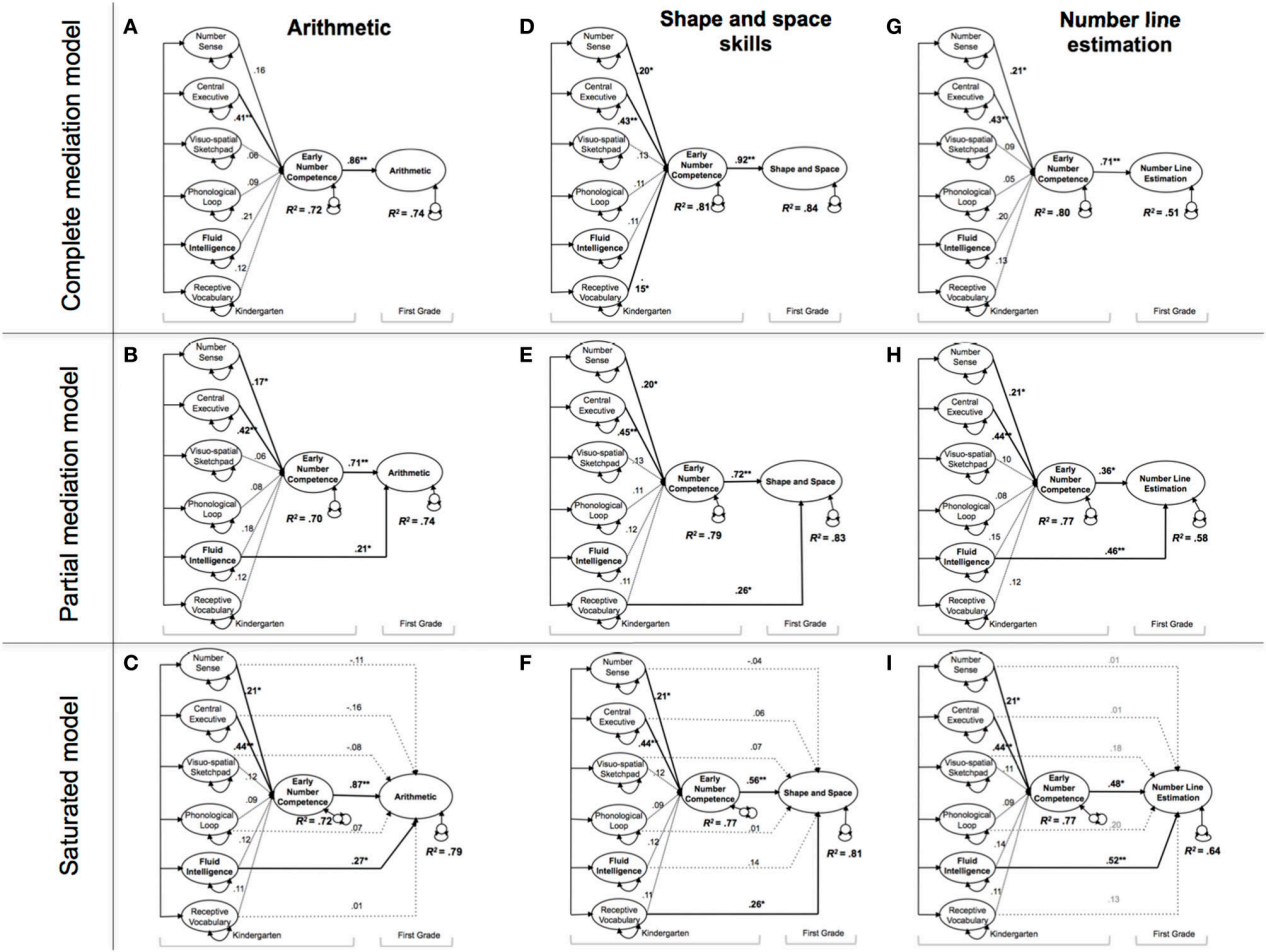
**The contributions of number-specific and domain-general abilities on first grade mathematics outcomes**. Nine mediation models to study how cognitive predictors measured at kindergarten influence math outcomes in first grade via early number competence. *N* = 165.

Figure 2 presents the nine mediation models; dashed lines indicate non-significant model parameters (*p* > 0.05). Each model was composed by a set of predictors measured at kindergarten (i.e., nonverbal number sense and domain-general abilities) that was specified to predict the mediator, ENC, that in turn predicted first grade math outcome.

We first turn to the relationship between basic cognitive abilities and ENC. As expected, these relationships were very similar in all estimated models and showed that nonverbal number sense and the central executive both uniquely contributed to ENC, which aligns with prior findings (Kroesbergen et al., 2009; Xenidou-Dervou et al., 2013). ENC was stronger related to the central executive (β = 0.44, *p* < 0.001) than to nonverbal number sense (β = 0.22, *p* < 0.05), suggesting that executive processes related to WM capacity are key domain-general mental resources for developing number skills such as counting in early childhood (cf. Noël, 2009). It is important to note that although nonverbal number sense and WM capacity were statistically the most significant contributors to ENC in all nine structural models, the latent correlations in Table 2 indicate that all the kindergarten measures were highly intercorrelated and correlated with ENC. These strong correlations suggest that number-specific, verbal and domain-general predictors share common cognitive processes and commonly explain a large part of ENC (across models the amount of explained variance in ENC ranged from *R*^2^ = 0.70 [Model b] to *R*^2^ = 0.81 [Model d]). Nonverbal number sense and central executive might the most important contributors because on the one hand the correlation between ENC and other basic cognitive abilities might be due to the variance that these abilities share with the central executive. Indeed the correlations between the central executive and other basic cognitive abilities are overall high (ranging from 0.44 to 0.67, see Table 2). On the other hand, number sense includes specific cognitive processes such as approximate quantity estimation that should be necessary beyond domain-general skills in order to develop ENC. The observed pattern of a large amount of explained variance in ENC in combination with significant regression weights of the central executive and nonverbal number sense and non-significant regression weights of the other cognitive predictors as found in all nine models, support this conclusion.

We now turn to the mediation analyses. Specifically, these analyses further revealed the processes involved in early math development and the relations between general and specific cognitive predictors and ENC measured in kindergarten, and specific math outcomes in first grade.

Models a, b and c tested the relations between basic predictors measured in kindergarten, ENC measured in kindergarten and first grade arithmetic. Model a was specified as a complete mediation model, hypothesizing perfect mediation by ENC, meaning the relations between basic predictors measured in kindergarten and arithmetic were completely mediated by ENC. Model b was specified as a partial mediation model where significant direct effects of cognitive predictors on arithmetic were added. Finally, Model c referred to the saturated structural model, the least restricted model allowing all predictors to directly and indirectly (via ENC) influence arithmetic. Chi-square difference tests indicated a significant difference between Model a and Model b (*p* < 0.04), in favor of Model b. This shows that the direct effect of fluid intelligence is statistically significant and leads to a significant improvement in model fit (Jöreskog, 1993). Further, there was no significant difference between Model b and Model c demonstrating that over and above the effect of fluid intelligence on arithmetic, no further direct effects need to be included. Thus, we will focus our discussion on the results obtained for Model b, as this model provides the best balance between model fit and parsimony. Note that we will apply this rationale for choosing a preferred model also to the remaining six models, Models d to i. Specifically, the results of Model b indicate that in addition to the strong effect of ENC on arithmetic (β = 0.71), fluid intelligence also directly affected this math outcome (β = 0.21, *p* < 0.05).

In Models d, e and f, we investigated the relations between the predictors measured in kindergarten, ENC and first grade shape and space skills. Similarly to the previous Models a, b and c, chi-square difference tests yielded a significant difference between the complete mediation model and the partial mediation model (*p* < 0.02), in favor again of the partial mediation model, Model e. Including an additional path between receptive vocabulary and shape and space skills significantly improved model fit. No significant difference was observed between Model e and Model f (the saturated model). We therefore retained Model e–the partial mediation model. In this model receptive vocabulary had an additional direct effect on shape and space skills (β = 0.26, *p* < 0.05) after controlling for ENC, the mediator (β = 0.72, *p* < 0.01). Both constructs explained 83 percent of the variance among first-graders performance in shape and space outcomes.

In Models g, h and i, we investigated the relations between basic predictors measured in kindergarten, ENC and first grade number line estimation. We observed a significant chi-square difference between the complete mediation and the partial mediation models (*p* < 0.01), in favor of the partial mediation model, Model h. There was no significant difference between Model h and Model i. We therefore retained Model h–the partial mediation model. In particular, in Model h fluid intelligence independently affected number line estimation (β = 0.46, *p* < 0.01) after accounting for ENC (β = 0.36, *p* < 0.05). Both constructs explained 58% of the variance among first graders performance in number line estimation.

To further substantiate the role of ENC as a critical mediator (as suggested by the three preferred partial mediation models–Models b, e, and h), we examined whether any cognitive predictors had a significant indirect effect on first grade math outcomes that is mediated via ENC (see Table 5).

**Table 5:** **Standardized indirect mediation effects, significance tests and bootstrapping confidence intervals for the three best-fitting SEM models**.

**Mediation effect**	**Standardized indirect effect^b^**	***SE***	***P***	**95%-CI**
**ARITHMETIC (MODEL b)**				
Number sense→ENC→Arithmetic	0.12	0.086	0.16	[−0.05 to 0.29]
Central executive→ENC→Arithmetic	0.30	0.096	0.01	[0.11 to 0.49]
Phonological loop→ENC→Arithmetic	0.06	0.068	0.39	[−0.08 to 0.19]
Visuo-spatial sketchpad→ENC→Arithmetic	0.04	0.073	0.57	[−0.10 to 0.18]
Receptive vocabulary→ENC→Arithmetic	0.09	0.069	0.21	[−0.05 to 0.22]
Fluid intelligence→ENC→Arithmetic	0.13	0.18	0.48	[−0.23 to 0.48]
**SHAPE & SPACE SKILLS (MODEL e)**				
Number sense→ENC→Shape & space skills	0.15	0.080	0.06	[−0.01 to 0.30]
Central executive→ENC→Shape & space skills	0.32	0.101	0.001	[0.12 to 0.52]
Phonological loop→ENC→Shape & space skills	0.08	0.071	0.28	[−0.06 to 0.22]
Visuo-spatial sketchpad→ENC→Shape & space skills	0.09	0.078	0.23	[−0.06 to 0.25]
Fluid intelligence→ENC→Shape & space skills	0.08	0.095	0.38	[−0.10 to 0.27]
Receptive vocabulary→ENC→Shape & space skills	0.08	0.070	0.28	[−0.06 to 0.21]
**NUMBER LINE ESTIMATION (MODEL h)^a^**				
Number sense→ENC→Number line estimation	0.08	0.060	0.21	[−0.19 to 0.04]
Central executive→ENC→Number line estimation	0.16	0.090	0.08	[−0.34 to 0.02]
Phonological loop→ENC→Number line estimation	0.03	0.045	0.54	[−0.12 to 0.06]
Visuo-spatial sketchpad→ENC→Number line estimation	0.04	0.043	0.39	[−0.12 to 0.05]
Receptive vocabulary→ENC→Number line estimation	0.04	0.042	0.32	[−0.12 to 0.04]
Fluid intelligence→ENC→Number line estimation	0.05	0.15	0.72	[−0.35 to 0.24]

ENC, early number competence. All p values and confidence intervals were computed by means of the bias-corrected bootstrap method (MacKinnon, 2008 as implemented in Mplus 5.2 Muthén and Muthén, 1998–2007).

a Regarding number line estimation, absolute values were used for standardized indirect effects to avoid negative values.

b Specific indirect effect of a certain cognitive precursor on a certain mathematics outcome that is mediated via ENC.

Interestingly, the central executive was the only predictor that had a significant indirect effect on arithmetic (standardized indirect effect = 0.30, *p* < 0.01) and shape and space skills (standardized indirect effect = 0.32, *p* < 0.001). Thus, these analyses support that ENC is indeed critical to mediate the effects of the central executive (i.e., WM) on arithmetic and shape and space skills 1 year later.

Taken together, the present results confirm both hypotheses formulated above. First nonverbal number sense and WM capacity (i.e., central executive) independently contributed to ENC. ENC in turn is the strongest predictor of math outcomes measured 1 year later in first grade. Second, ENC takes the role of a critical mediator of the effects of WM capacity on arithmetic and shape and space skills in first grade.

## Discussion

The first aim of this study was to provide new insights in the developmental trajectories of children's math achievement in kindergarten and first grade. Therefore, we adopted a longitudinal approach and studied which basic cognitive abilities uniquely predicted math achievement in kindergarten and 1 year later in first grade. Drawing on structural equation modeling and mediation analyses we sought to provide a clearer view on the complex relationships among these various basic and acquired abilities.

Our results indicate that nonverbal number sense and WM capacity jointly contributed to ENC in kindergarten. The significant contribution of nonverbal number sense to ENC when controlling for WM highlight the importance of an approximate number system in the development of symbolic number skills in kindergarten. Similar findings have been reported recently (Starr et al., 2013; Xenidou-Dervou et al., 2013) and extend prior research that stressed the role of executive functions and WM in math development but did not control for nonverbal number sense (e.g., Espy et al., 2004; Noël, 2009).

### The contribution of WM

One explanation why WM substantially contributed to ENC is that ENC was predominantly measured by counting tasks drawing on attentional and executive processes. Verbal counting and enumeration tasks involve similar processes than typical serial span tasks measuring WM that require the constant updating of information such as keeping track of the verbal number sequence, remember which items have been counted and which items still need to be counted, and hence involve attentional processes. Tuholski et al. (2001) found that adult participants with lower WM capacity took longer to count vertical bars on a black screen than did those with higher WM capacity. By contrast, WM capacity groups did not significantly differ in subitizing—a highly automated process that is not dependent on controlled processing. Hence, differences emerged only during counting because attention had to be controlled over longer intervals.

While Jordan et al. (2010) found no independent contribution of WM to first grade math achievement after controlling for ENC, our structural mediation analyses, however, indicated that WM had significant indirect mediation effects on arithmetic and shape and space skills (also see Hassinger-Das et al., 2014). We therefore suggest that WM has a central role when children start to learn math in kindergarten and indirectly influences formal math achievement in first grade. Likewise, Imbo and Vandierendonck (2007) found variations in the relation between WM and arithmetic across developmental stages. The importance of WM decreased when children had developed more efficient strategies such as memory fact retrieval and factual knowledge. Good WM may then foster ENC and indirectly math achievement, whereas poor WM may delay its development and consequently indirectly slow growth in later math achievement (also see Noël, 2009). Rousselle and Noël (2007) found that seven-year-old dyscalculic children performed lower on Arabic number comparisons than typically developing children resulting from a general transcoding deficit (also see Defever et al., 2013). Transcoding deficit means that a child encounters difficulties in accessing quantity representations from abstract symbols. Since WM is responsible for storing, processing, retrieving and combining relevant information to yield new meaningful representations, which here imply to meaningful quantitative representations. This deficit may then be related to poor WM.

### The contribution of nonverbal number sense

Significant associations between nonverbal number sense and arithmetic performance have been presented in recent cross-sectional and longitudinal studies (in children: Libertus et al., 2011, 2012; Lonnemann et al., 2013; Xenidou-Dervou et al., 2013; in adults, DeWind and Brannon, 2012). Still, Xenidou-Dervou and colleagues' findings emphasize that the relationship between nonverbal number sense and counting skills was completely mediated by symbolic approximation skills in preschoolers. Likewise our results underscore the unique role of nonverbal number sense in developing number skills in kindergarten. Nonverbal number sense appears to play a critical role in the acquisition of early informal symbolic number knowledge (see also Starr et al., 2013). Similarly to Bartelet and colleagues, the present study found that the relationship between kindergarteners' nonverbal number sense and math achievement in first grade was completely mediated by early symbolic number skills. Bartelet et al. (2014) longitudinal study indicates that nonverbal number sense is not a critical predictor for first grade arithmetic fluency after controlling for symbolic number skills such as dot counting and Arabic number comparisons. Accordingly, they conclude that symbolic number skills are crucial predictors of early arithmetic performance.

Possibly nonverbal number sense did not significantly contribute to first grade math after controlling for WM capacity and ENC because kindergarteners' nonverbal number sense might be less precise than in older children who experienced formal education and developed symbolic number representations that in turn may sharpen approximate number representations (cf. Lonnemann et al., 2013; Piazza et al., 2013). From this rationale young children may use domain-general cognitive resources to a higher extent than older children who use more specific number knowledge (e.g., more precise number representations; number fact retrieval) when completing calculations.

### Predictors for first grade math achievement

Nonverbal number sense and WM turned out to be critical for ENC in kindergarten, but not for first grade math achievement after ENC was taken into account. This is due to the fact that math outcomes in first grade were strongly predicted by ENC. We therefore suggest that ENC is indeed a critical mediator in the relationship between kindergarten domain-specific and domain- general predictors and first grade math outcomes. Children who start first grade with advanced counting, enumeration and symbolic number comparison skills will consequently progress faster in arithmetic and generally in symbolic number tasks in first grade (also see Bartelet et al., 2014).

Over and above the effect of ENC on first grade math outcomes, this study provided evidence for a unique contribution of fluid intelligence on arithmetic and number line estimation on the one hand and for a unique contribution of receptive vocabulary to shape and space skills. Although first grade arithmetic builds mainly on ENC, Model b indicated that fluid intelligence additionally explained arithmetic performance. One reason might be that children's fluid intelligence –the ability to induce and apply rules and procedures- is required to solve arithmetic operations at least at an initial stage at first grade. According to this view, first grade arithmetic draws on symbolic number skills and reasoning ability and hence should be considered a more complex competence than ENC Likewise, kindergarteners' fluid intelligence also directly predicted their number line estimation in first grade. Accurate number line estimation appears to depend on a good understanding of the place value system of our numbers that might draw on higher-order thinking skills such as reasoning ability early in math development when symbolic number skills and number routines are less developed. Likewise, Geary (2013) claims that intelligence fosters early explicit number system knowledge and the comprehension of procedures that can be used to operate on numbers. Accordingly, the finding that fluid intelligence was a stronger predictor for number line performance than ENC suggests that the children in our sample were still learning to understand the logical structure of the number line. Once children have acquired this logical systematic structure, fluid intelligence becomes less important for number-line performance (Geary, 2013).

The additional contribution of receptive vocabulary to shape and space skills suggests that children's verbal comprehension ability partly explained shape and space skill performance. Children were less familiar with both tasks assessing shape and space skills and first had to comprehend the task instructions in order to complete the tasks. Moreover, the task instructions on these tasks were a little longer than on the other math tasks. As a result, it is likely that good verbal comprehension facilitated the understanding and completion of both shape and space tasks. This might have specifically affected children with migration background who show overall lower verbal comprehension abilities (EMACS, 2012). Even though there is a current trend for teaching problem solving and solving strategies in first grade, the national curriculum as well as national math textbooks primarily focused until lately on arithmetic competencies in first grade. Thus, children from the present sample were less habituated to work on tasks requiring some form of problem solving or deducing original solving strategies, which might also explain the unique contribution of verbal skills on first grade math outcomes.

### Limitations

Several limitations of the present study warrant consideration. First, basic cognitive abilities including nonverbal number sense, WM components, fluid intelligence, receptive vocabulary, and ENC were concurrently assessed at the end of kindergarten. The causal relationships tested between ENC and cognitive predictors therefore reflect the theoretical position that ENC is a complex construct drawing on multiple cognitive processes, both number-specific and domain-general. Future studies assessing children's basic cognitive and number-specific processes at several measurement points may provide further insights into the foundation of ENC.

Second, although the present sample size was large for developmental studies in preschool age, the statistical power associated with this sample size may not have been sufficient to detect small indirect effects of cognitive predictors on math outcomes in addition to those observed for WM capacity. Future studies may therefore benefit from drawing on larger sample sizes to study the mediational power of ENC.

Third, our assessment of ENC predominantly focused on tasks requiring procedural counting knowledge (except the Arabic number comparison task, which requires place-value knowledge). For 5-year-old children, completing such procedures is effortful, not yet automatic, and therefore highly dependent on WM processes. In future, tasks measuring number-specific conceptual knowledge (e.g., estimate the correctness of counting procedures) should be included in early math batteries since this type of tasks is less dependent on conscious attention and cognitive resources (also see Schneider and Stern, 2010). To date, however, procedural knowledge remains fundamental to early math learning in formal school settings from childhood to adulthood (Floyd et al., 2003) and may in some contexts support conceptual learning (Canobi and Bethune, 2008).

Fourth, we did not assess processing speed in kindergarten. Prior studies showed that processing speed is related to early math development (Passolunghi and Lanfranchi, 2012). Thus, we urge future research to include processing speed measures when studying the relationships between basic cognitive predictors and math outcomes.

### Implications for future research

According to prior findings (Koponen et al., 2007; Kroesbergen et al., 2009) and suggestions (De Smedt et al., 2009), multiple cognitive components need to be taken into account when examining the processes underlying the multifaceted constructs of ENC and later math achievement (also see Fuchs et al., 2010b). We have sought to cover a broad range of such processes in this study. Future research should continue investigating the relations of potentially important basic cognitive abilities with math achievement from a longitudinal perspective. For example, finger counting and finger gnosia are thought to play a particular role in basic arithmetic (Carlson et al., 2007; Kaufmann, 2008; Crollen et al., 2011). It would therefore be interesting to extend the present study by assessing and including children's problem solving strategies in math tasks such as the use of hands (also see Schneider et al., 2009 for applied strategies in the number line estimation task).

### Implications for assessment and educational practices

In conclusion, we firmly believe that the insights provided by broad-based longitudinal studies into the cognitive underpinnings of early number and later math development will directly benefit the teaching practice and development of early screening and intervention programs. Children from underprivileged families may particularly benefit from everyday numeracy activities in early education (e.g. Starkey et al., 2004). More generally, targeted early educational support programs may promote later math achievement and thus help to reduce learning gaps between children from different social backgrounds and with different cognitive abilities (Caro et al., 2009). On the one hand we suggest that interventions should include number-specific tasks to train number-related procedures and strategies and to foster the understanding of numbers and their relations. Several recent number-specific training studies have produced promising results to support children with symbolic math difficulties (Wilson et al., 2009). Recently, Park and Brannon (2013) showed that the training of adults' approximate arithmetic skills improved their symbolic arithmetic performance. On the other hand, recent intervention studies have shown that poor WM can be enhanced, with potentially positive effects on children's ENC (Kroesbergen et al., 2014) and math learning (Klingberg et al., 2005; Holmes et al., 2009). Further research on the long-term benefits of different types of intervention programs on math development in children “at-risk” might prove fruitful to help define how these different intervention forms should be scheduled and combined.

## Conclusion

The major findings are that (a) nonverbal number sense and WM capacity independently contributed to ENC in kindergarten, (b) ENC was the strongest predictor for arithmetic and shape and space skills in first grade, (c) WM capacity had an indirect effect on arithmetic and shape and space skills in first grade, (d) fluid intelligence independently predicted arithmetic and number line estimation after controlling for ENC, and (e) receptive vocabulary independently predicted shape and space skills after controlling for ENC. In line with LeFevre et al. (2010), we conclude that specific math outcomes draw to unequal extents on different constellations of domain-specific and domain-general processes.

In sum, our findings emphasize both the direct and indirect contributions of domain-general abilities (fluid intelligence and executive processes of WM) after accounting for symbolic number skills when performing formal math tasks in first grade involving number combinations, place value knowledge and task comprehension.

The current study extends prior findings by including a broad range of plausible cognitive predictors to explain math outcomes 1 year later and by testing a comprehensive early math development model using SEM and mediation analyses. The role of ENC for first grade math is indeed critical. Children with lower ENC showed lower arithmetic performances 1 year later. This result gives clear directions for targeted intervention to improve children's ENC already in kindergarten before formal instruction and consequently boost later math achievement

## Authors note

Caroline Hornung, Christine Schiltz and Romain Martin designed the study. Caroline Hornung collected the data, prepared the data for analyses, and wrote the manuscript. Caroline Hornung, Christine Schiltz, Martin Brunner and Romain Martin analyzed and interpreted the data. Caroline Hornung, Christine Schiltz, Martin Brunner and Romain Martin critically revised the manuscript and approved the final version to be published.

### Conflict of interest statement

The authors declare that the research was conducted in the absence of any commercial or financial relationships that could be construed as a potential conflict of interest.
