# Supplementary material for: Predicting first-grade mathematics achievement: the contributions of domain-general cognitive abilities, nonverbal number sense, and early number competence
Source: Front Psychol. 2014 Apr 4;5:272. doi: 10.3389/fpsyg.2014.00272 (PMC3983481; doi:10.3389/fpsyg.2014.00272)
Supplement: Supplementary file 1 [file Presentation1.PDF]

Appendix A

Cognitive Tests Administered, with Subtests and Example Items

<table>
<tr><th></th><th>Construct</th><th>Subtest</th><th>Content and example of items</th></tr>
<tr><td rowspan="16">Administered in kindergarten</td><td rowspan="6">Early number competence (ENC)</td><td rowspan="4">Verbal counting scale</td><td>Count up to 50</td></tr>
<tr><td>Count up from 8</td></tr>
<tr><td>Count backwards from 23</td></tr>
<tr><td>Count 2 steps from 5</td></tr>
<tr><td>Dot counting</td><td>Count an array of six dots aligned</td></tr>
<tr><td>Arabic number comparison</td><td>Which number is the largest,
5 or 9?</td></tr>
<tr><td rowspan="6">Working memory (WM)</td><td rowspan="2">Phonological loop: Verbal forward span</td><td>Digit recall
6 9 4 - 6 9 4</td></tr>
<tr><td>Pseudoword recall
MA LU PI - MA LU PI</td></tr>
<tr><td rowspan="2">Central executive: Backward span</td><td>Backward Digit span
8 1 4 - 4 1 8</td></tr>
<tr><td>Backward Colour span
gréng giel blo - blo giel gréng
(i.e., green yellow blue)</td></tr>
<tr><td rowspan="2">Visuo-spatial sketch pad:
Location span</td><td>Free recall of a sequence of 2, 3, or 4 positions of a dwarf on a screen with a grid</td></tr>
<tr><td>Free recall of a sequence of 2, 3, or 4 positions of a dwarf on a screen without a grid</td></tr>
<tr><td rowspan="2">Nonverbal number sense (NS)</td><td rowspan="2">Approximate numerosity comparison</td><td>Dots comparison
6 vs. 8 dots</td></tr>
<tr><td>Sticks comparison
16 vs. 20 sticks</td></tr>
<tr><td>Receptive Vocabulary (RV)</td><td>British Picture Vocabulary Scale</td><td>4 pictures of animals are shown and the child is asked to point to the cat</td></tr>
<tr><td>Fluid Intelligence (GF)</td><td>Raven's Coloured Progressive Matrices</td><td>The child identifies the missing piece needed to complete a pattern.</td></tr>
</table>

<table>
<tr><td rowspan="3">Administered in first grade</td><td rowspan="3">Math Outcomes</td><td rowspan="3">Arithmetic</td><td>Addition
1 + 6 = __</td></tr>
<tr><td>Subtraction
4 – 1 = __</td></tr>
<tr><td>Basic equations
__ + 4 = 8</td></tr>
</table>

			Number comparisons<br>(< > =)<br>11 ___ 12
			Number sequence<br>2 4 6 8 __ __ __<br>The child has to identify the rule or pattern behind a sequence of numbers.
		Shape & space skills	Length estimation<br>The child estimates the length of a set of lines by comparing them with three lines representing lengths of 1, 5 and 10 steps, respectively.
			Counting Blocks<br>The child has to find out how many cubes constitute a shape. The difficulty is that not all cubes are directly visible.
		Number line estimation	The child places numbers on a physical number line from 0 to 100, (e.g., 19).
